# Supplementary material for: Mitophagy Regulates the Circadian Rhythms by Degrading NR1D1 in Simulated Microgravity and Isolation Environments
Source: Int J Mol Sci. 2024 Apr 29;25(9):4853. doi: 10.3390/ijms25094853 (PMC11084518; doi:10.3390/ijms25094853)
Supplement: Supplementary file 1 [file ijms-25-04853-s001.zip › ijms-2928903-supplementary.pdf]

Supplementary Materials

Mitophagy Regulates the Circadian Rhythms by Degrading NR1D1 in Microgravity and Isolation Environments

Sihai Zhou [1,2,†], Xiaopeng Li [2,†], Fengji Liang [2], Guohua Ji [2], Ke Lv [2], Yanhong Yuan [2], Yujie Zhao [2], Na Yan [2], Chuanjie Zhang [2], Shiou Cai [2], Shuhui Zhang [2], Xu Liu [2], Bo Song [1,*] and Lina Qu [2,*]

1 Department of Pathology and Forensics, Dalian Medical University, Dalian 116044, China; zshdoct@163.com (S.Z.)

2 State Key Laboratory of Space Medicine, China Astronaut Research and Training Center, Beijing 100000, China. lixiaopeng_acc@163.com (X.L.); freejohnnyliang@126.com (F.L.); jgh1682004@126.com (G.J.); lvke_med@aliyun.com (K.L.); yyh_yuan@163.com (Y.Y.); zhaoyujie9797@163.com (Y.Z.); yanna202204@163.com (N.Y.); zhangchuanjie96@163.com (C.Z.); caishiou19910228@163.com (S.C.); sherlyhuihui@sina.com (S.Z.); xuxu_lh@163.com (X.L.)

* Correspondence: bosong@dmu.edu.cn (B.S.); linaqu@263.net (L.Q.)

† These authors contributed equally to this work.

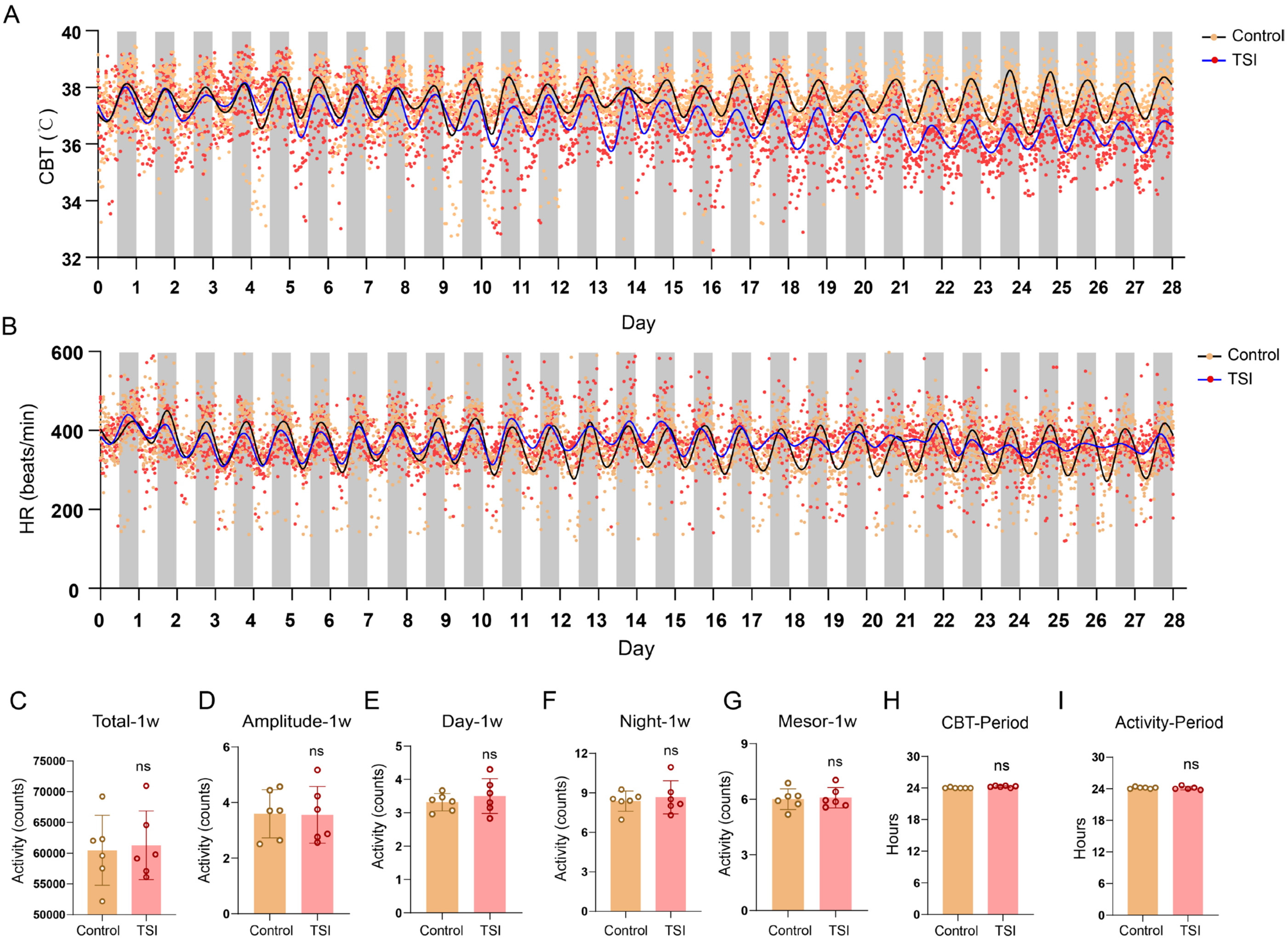

Supplementary Figure S1. TSI induces the disturbance of circadian rhythms in rats. (**A, B**) The curve fitting of the CBT (**A**) or **HR** (**B**) data of rats during the 28-day period from control and TSI groups (Each point represents the average CBT or HR values corresponding to each hourly time point from control and TSI rats). (**C-G**) Analysis of total (**C**), amplitude (**D**), daytime (**E**), nighttime (**F**) and mesor (**G**) of the locomotor activity data during the initial 7-day period. (**H**) Analysis of period (tau) of the CBT data during the final 7-

day period. (**I**) Analysis of period (tau) of the HR data during the final 7-day period. n = 6. ns: none significance. Two-sided Student’s t tests were used to measure the significance.

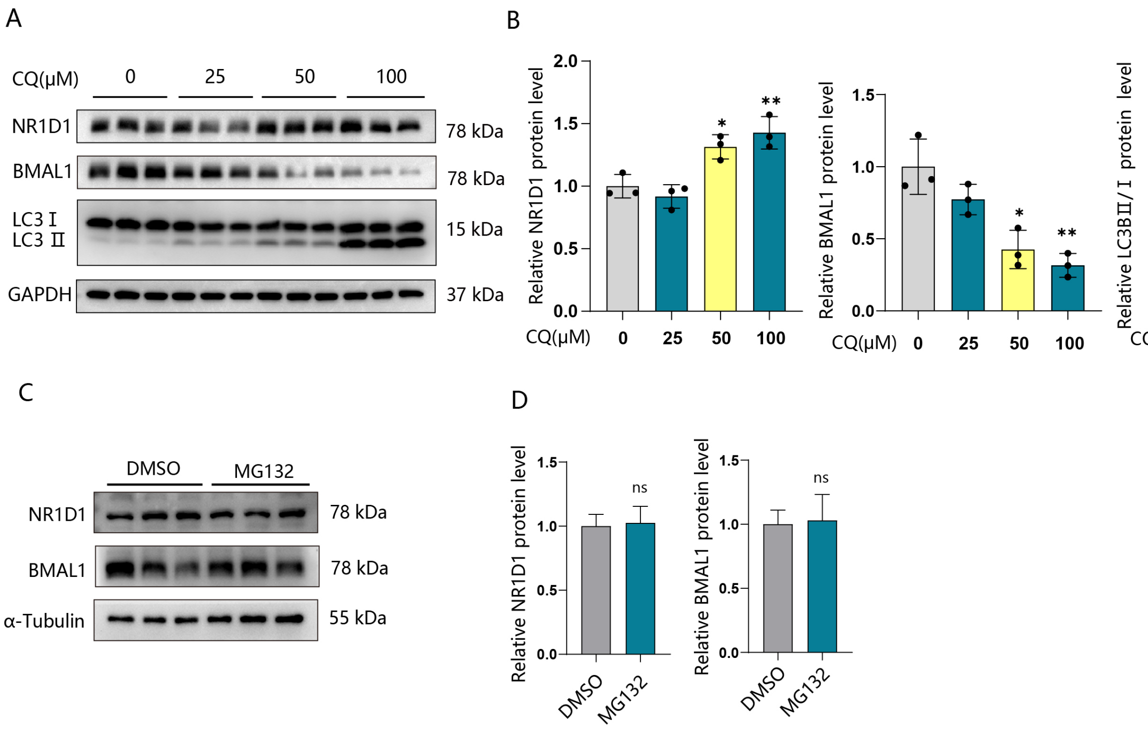

Supplementary Figure S2. NR1D1 is degraded by autophagy. (**A**) Western blotting analysis of the protein NR1D1, BMAL1 and LC3 after using CQ (25 μM, 50 μM, 100 μM or vehicle) in NIH3T3 cells for 24 h. (**B**) Quantification of NR1D1, BMAL1 and LC3 Ⅱ/Ⅰ protein expression (n = 3). (**C**) Western blotting analysis of the protein NR1D1, BMAL1 after treating with MG132 (5 μM) in NIH3T3 cells for 24 h. (**D**) Quantification of NR1D1, BMAL1 protein expression (n = 3). *$p < 0.05$; **$p < 0.01$; ***$p < 0.001$. ns: none significance. Data are mean ±S.E.M.. Two-sided Student's t tests were used to measure the significance.

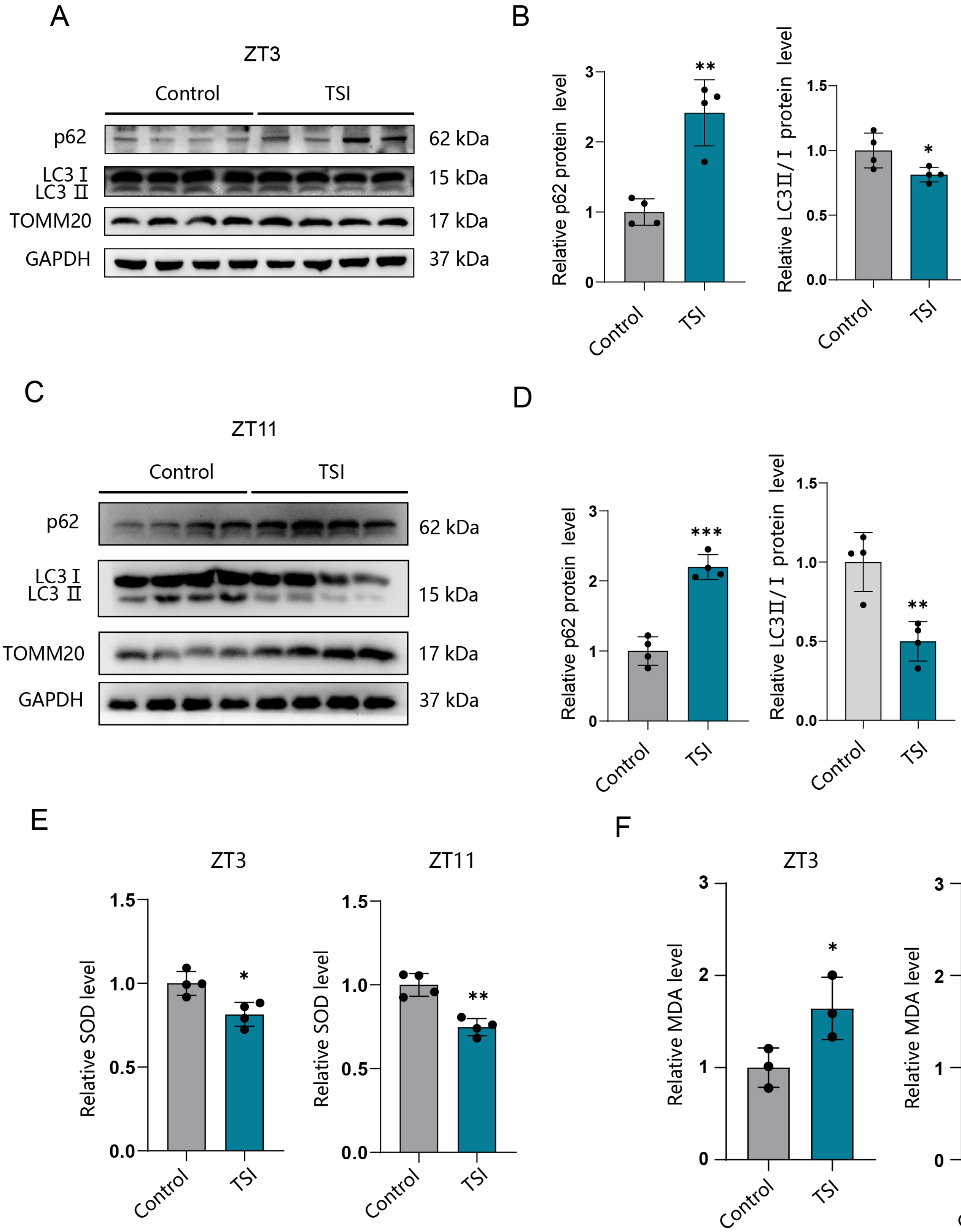

Supplementary Figure S3. (**A**) The TSI environment causes mitophagy deficiency and mitochondrial dysfunction in neurons of the SCN. Western blotting analysis of the protein p62, LC3 Ⅱ/Ⅰ and TOMM20 of the SCN from control and model groups at ZT3 (n = 4). (**B**) Quantification of p62, LC3 Ⅱ/Ⅰ and TOMM20 protein expression at ZT3 (n = 4). (**C**) Western blotting analysis of the protein p62, LC3 Ⅱ/Ⅰ and TOMM20 of the SCN from control and model groups at ZT11 (n = 4). (**D**) Quantification of p62, LC3 Ⅱ/Ⅰ and TOMM20 protein expression at ZT11 (n = 4). (**E-F**) Quantification of the activity of SOD (**E**) and MDA (**F**) in the SCN tissues from control and model groups at ZT3 and ZT11. *p < 0.05; **p < 0.01; ***p < 0.001. Data are mean ± S.E.M., n = 4. Two-sided Student's t tests were used to measure the significance.

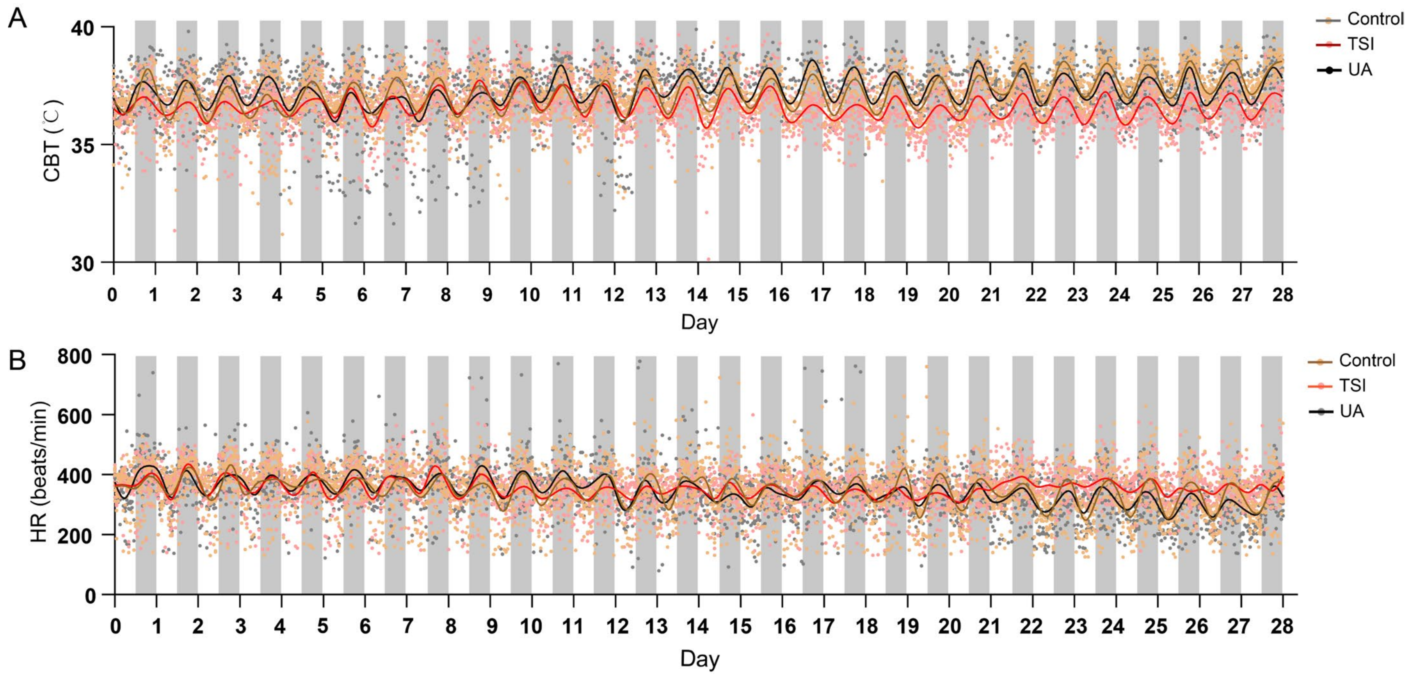

Supplementary Figure S4. UA Ameliorates the Disturbance of SCN Rhythms. (**A, B**) The curve fitting of the CBT (**A**) or HR (**B**) data of rats during the 28-day period from control, TSI and UA-treated groups (Each point represents the average CBT or HR values corresponding to each hourly time point from control, TSI and UA-treated rats). n = 6. Data are mean ± S.E.M.. Two-sided Student's t tests were used to measure the significance).

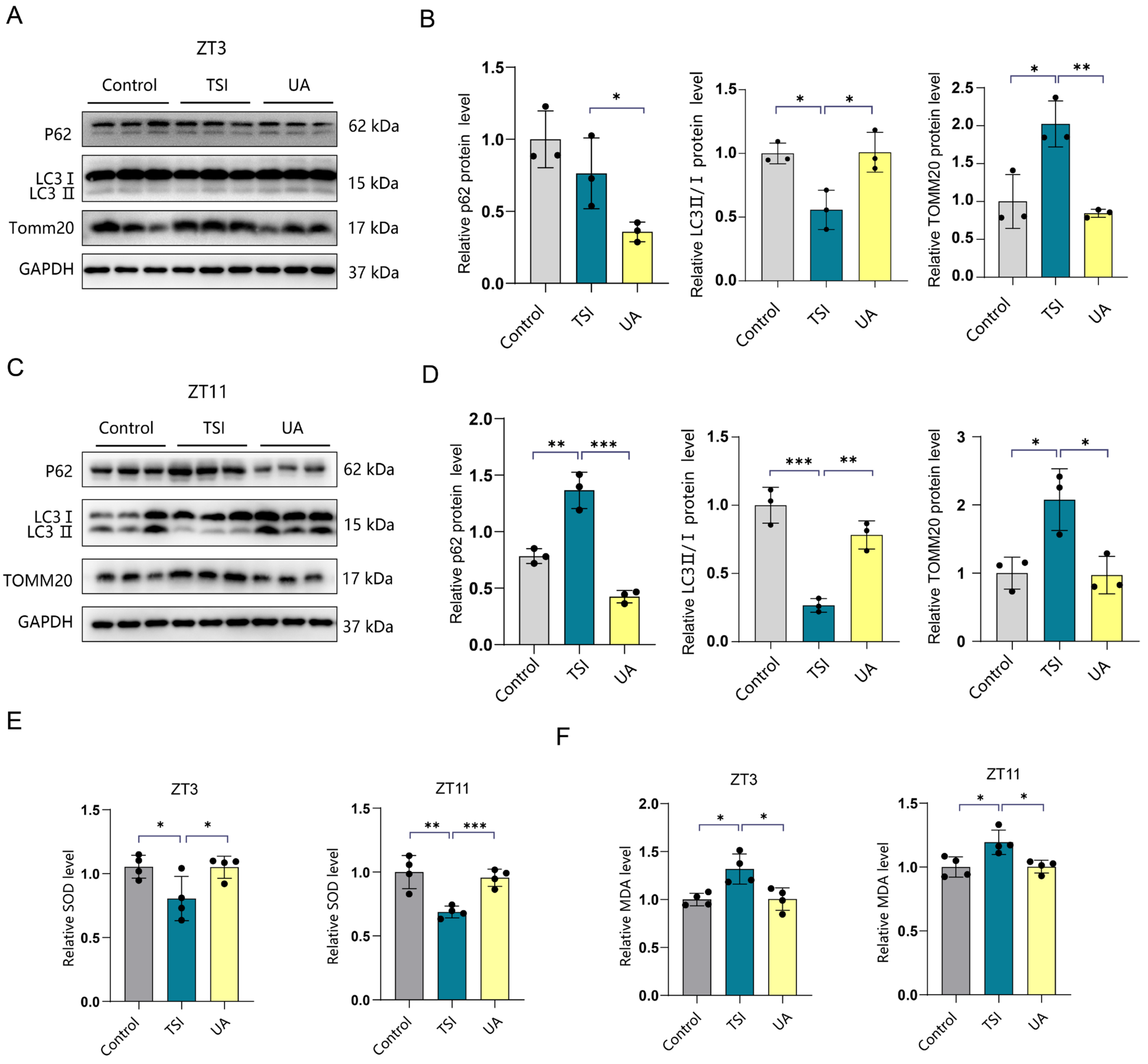

Supplementary Figure S5. UA ameliorates the disturbance of circadian rhythms through promote the degradation of NR1D1 in rats. (**A, C**) Western blotting analysis of the protein p62, LC3 II/I and TOMM20 of the SCN from TSI and UA-treated group (n = 3) at ZT3 and ZT11. (**B, D**) Quantification of the normalized p62, LC3 II/I and TOMM20 protein expression at ZT3 (n = 3) at ZT3 and ZT11. (**E, F**). Quantification of the activity of SOD (**E**) and MDA (**F**) in the SCN tissues from control and model groups at ZT3 and ZT11 (n = 4). ns: none significance. *p < 0.05; **p < 0.01; ***p < 0.001. Data are mean ± S.E.M.. Two-sided Student's t tests were used to measure the significance.
